# The utility of synoptic operation reports in colorectal surgery: a systematic review

**DOI:** 10.1007/s00384-024-04613-y

**Published:** 2024-04-30

**Authors:** Amanda Nikolic, Isaac Tranter-Entwistle, Andrew McCombie, Saxon Connor, Tim Eglinton

**Affiliations:** 1 Te Whatu Ora, Waitaha, Christchurch, New Zealand; 2 Department of Surgery and Critical Care, University of Otago, Christchurch, New Zealand; 3 Christchurch Hospital, C/O Department of Surgery, 2 Riccarton Avenue, Christchurch Central City, Christchurch, 4710 New Zealand

**Keywords:** Surgeons, Colorectal neoplasms, Electronic health records, Documentation

## Abstract

**Purpose:**

Accurate documentation is crucial in surgical patient care. Synoptic reports (SR) are structured checklist-based reports that offer a standardised alternative to traditional narrative reports (NR). This systematic review aims to assess the completeness of SR compared to NR in colorectal cancer (CRC) surgery. Secondary outcomes include the time to completion, surgeon satisfaction, educational value, research value, and barriers to implementation.

**Methods:**

Prospective or retrospective studies that assessed SR compared to NR in colorectal cancer surgery procedures were identified through a systematic search of Ovid MEDLINE, Embase (Ovid), CIHNAL Plus with Full Text (EBSCOhost), and Cochrane. One thousand two articles were screened, and eight studies met the inclusion criteria after full-text review of 17 papers.

**Results:**

Analysis included 1797 operative reports (NR, 729; SR, 1068). Across studies reporting this outcome, the completeness of documentation was significantly higher in SR (*P* < 0.001). Reporting of secondary outcomes was limited, with a predominant focus on research value. Several studies demonstrated significantly reduced data extraction times when utilising SR. Surgeon satisfaction with SR was high, and these reports were seen as valuable tools for research and education. Barriers to implementation included integrating SR into existing electronic medical records (EMR) and surgeon concerns regarding increased administrative burden.

**Conclusions:**

SR offer advantages in completeness, data extraction, and communication compared to NR. Surgeons perceive them as beneficial for research, quality improvement, and teaching. This review supports the necessity for development of user-friendly SR that seamlessly integrate into pre-existing EMRs, optimising patient care and enhancing the quality of CRC surgical documentation.

## Introduction

Accurate documentation is essential for ensuring patient safety and quality of care in surgery. Traditionally, narrative operation reports (NR) have been used to record operative procedures [1]. However, these reports have several drawbacks, including subjectivity, inconsistency, incompleteness and difficulties in interpretation [2, 3]. This has implications for the individual operative case and precludes ready analysis and utility in research and audit [2, 4].

A promising alternative to NRs is the use of synoptic operative reports (SR), also known as structured or checklist-based reports. SRs utilise templates to provide a standardized approach to surgical documentation [1, 2]. Surgeons can capture the essential data points in a concise and organised manner using standardised terminology [1, 2]. This format allows for easy retrieval of data for analysis.

Synoptic reporting has been successfully implemented in various aspects of colorectal cancer (CRC) care, such as MRI and pathology reports [5, 6]. It has also shown utility in other surgery types [3, 7, 8]. The benefits of SR include increased completeness, capturing of quality measures, reduced documentation time, and serving as an educational tool for surgeons and trainees [1, 3, 8–11]. Additionally, the prospective collection of standardised operative data through synoptic reports is valuable for research and driving process change [11, 12].

Colorectal surgery is a complex and diverse field that requires accurate and comprehensive documentation for optimal patient care and to facilitate research. Before implementing SR, it is crucial to define their potential benefits in CRC surgery and identify barriers to their development and implementation.

This systematic review aims to comprehensively assess the role of SR in CRC surgery. The primary objective is to evaluate the completeness of SR compared to NR in documenting CRC procedures. Secondary outcomes, including surgeon satisfaction, and the educational and research value of SR, will be critically appraised. Furthermore, this study will assess the challenges associated with implementing SR systems and provide insights into potential solutions.

## Methods

### Protocol and registration

This systematic review was conducted and reported in line with PRISMA (Preferred Reporting Items for Systematic Reviews and Meta-Analyses) [13] and AMSTAR (Assessing the methodological quality of systematic reviews) guidelines [14]. The protocol was registered with the International Prospective Register of Systematic Reviews (PROSPERO, http://www.crd.york.ac.uk/prosper) before the start of the systematic review, with registration number CRD42023400109.

### Eligibility criteria

Included in this review were prospective and retrospective studies that assessed the performance of narrative operative notes compared to synoptic operative notes for colorecta**l cancer surgery procedures were included. NR referred to traditional or dictated notes without a formal structure, while SR involved the use of standardized reporting elements in structured reports. Studies evaluating primary or secondary outcome measures were included.

### Outcome measures

The primary outcome measure was the completeness percentage of operative reports, focusing on the collected variables in each study. Secondary outcomes included time to completion, surgeon satisfaction, barriers to implementation, educational value, and research utility.

### Search strategy

Researchers conducted a systematic search of four databases: Ovid MEDLINE, Embase (Ovid), CIHNAL Plus with Full Text (EBSCOhost), and Cochrane. Search terms combining operation notes AND colorectal surgery types were used, incorporating subject headings and keywords in titles or abstracts. Boolean operators AND/OR connected search terms. The search strategy was initially developed for MEDLINE (Supplemental Digital Content 1) and then adapted for the relevant databases. Studies published from 1990 onwards were included, while non- English publications were excluded. Additionally, the reference lists of randomised controlled trials (RCTs), meta-analysis, and systematic reviews were manually searched for potentially relevant studies. The searches were performed on 20th of February, 2023. Search records were imported into Endnote, and duplicates were removed. Covidence systematic review software was used for further screening (Covidence systematic review software, Veritas Health Innovation, Melbourne, Australia).

### Data extraction

Two independent reviewers (AN and ITE) conducted an initial screening of records based on titles and abstracts. Cohen’s kappa statistic for inter-rater reliability (IRR) demonstrated substantial agreement between the reviewers (IRR = 0.82) [15]. The remaining records underwent full-text screening using eligibility criteria (IRR 0.77). Any disagreements were resolved through discussion and consensus.

A single researcher (AN) used a standard data template to extract data related to primary and secondary outcomes of this review.

### Risk of bias

Due to the absence of randomised controlled trials in the extracted studies, a risk of bias assessment tool was not employed.

### Data analysis

Meta-analysis was not feasible with the available primary and secondary outcome data; thus, a narrative summary was provided.

## Results

**Fig. 1:**
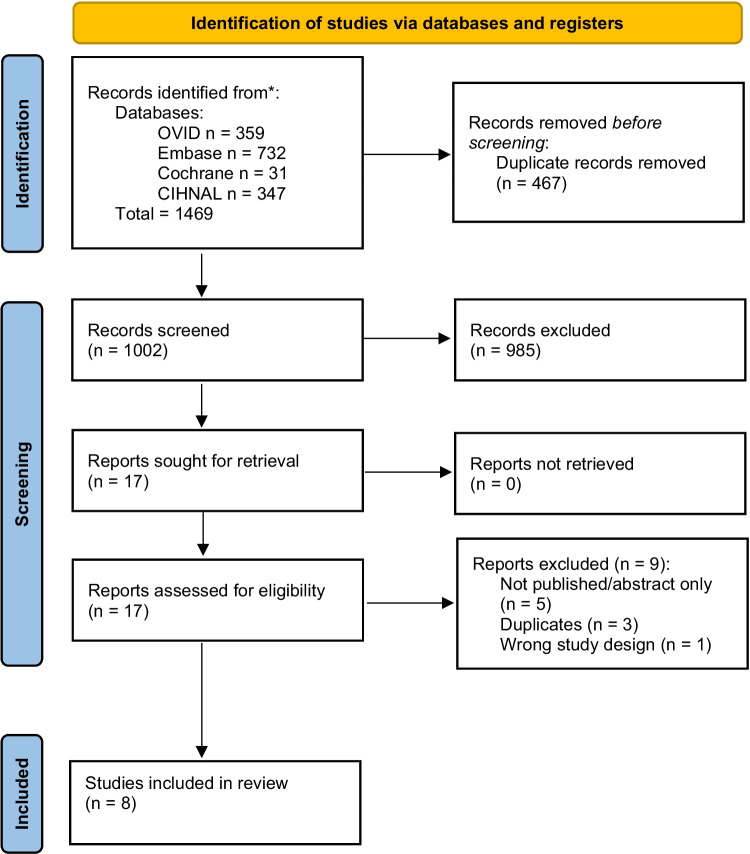
PRISMA flowchart

A total of 1002 articles were screened after removing duplicates using titles and abstracts. This resulted in 17 full-text reviews, leading to the inclusion of eight studies in the final analysis (Fig. 1).

### Study characteristics

The analysis included 1797 operative reports, comprising 729 NR and 1068 SR. Table 1 provides detailed information about the included studies. Six studies focused on SR implementation in rectal cancer resections [3, 4, 7, 12, 16, 17], one study on colon cancer resections [2], and one study assessed both types [2]. Except for Bidwell et al. [16], all studies evaluated the completeness of surgical reports as the primary outcome, comparing them to a set of defined variables. The secondary outcomes, such as time to completion, surgeon satisfaction, barriers to implementation, and educational and research value, varied across the studies.

**Table 1:** Summary of included studies and their characteristics

Author (year)	Procedure	Study type	Type of narrative report	Type of synoptic report	Narrative reports (number)	Synoptic reports (number)	Primary outcome	Secondary outcome
Kanters 2020 [4]	Rectal cancer resections	Prospective observational	Narrative only report	Any synoptic template included within the report	48	62	Inclusion of key reporting elements recommended by the National Accreditation Program for Rectal Cancer (same as the ASCRS^b^ rectal cancer checklist)	-
Bidwell 2020 [7]	Rectal cancer resections	Prospective SR data collection compared to retrospectively collected NR. Convergent mixed-methods design	Narrative report	Synoptic report based on ASCRS^b^ rectal cancer checklist	180	118	Change in fidelity to documentation of 19 critical items as defined by ASCRS^b^ rectal cancer checklist	Details and perspectives from surgeons about SR
Buchanan 2022 [2]	Colon and rectal cancer resections	Retrospective observational	Narrative report	Synoptic report developed by adapting key colon and rectal cancer-specific quality measures from BCCA^a^ database and consensus between colorectal surgeons	104	491	Reporting of colon and rectal cancer specific quality measures as developed by adapting key colon and rectal cancer-specific quality measures from BCCA^a^ database and consensus between colorectal surgeons	Synoptic report use
Maniar 2014 [18]	Colon cancer resections	Prospective SR data collection Retrospective historical NR cohort	Narrative report	WebSMR^c^ generated synoptic note	80	80	Reporting of a list of quality indicators extracted from two published articles ^25,26^, excluding indicators specific to rectal cancer surgery	Interrater agreement and time to extract data
Maniar 2015 [3]	Rectal cancer surgery	Prospective SR data collection Retrospective historical NR cohort	Narrative report	Synoptic report developed using the ASCRS^b^ rectal cancer checklist	97	97	Reporting of a list of quality indicators defined by the ASCRS^b^ rectal cancer checklist	Time to abstract data from reports and inter-rater reliability
Bidwell 2022 [16]	Rectal cancer resections	Prospective SR data collection and compared to a retrospective NR. Prospective mixed-methods study using surveys and qualitative interviews	Narrative report	Synoptic report developed using the ASCRS^b^ rectal cancer checklist, colorectal surgery experts and key stakeholder involvement. 19 items	140	140	Acceptability, feasibility, usability. Experiences with implementation and motivations and barriers to use	
Edhemovic 2004 [12]	Rectal cancer resections	Prospective SR data collection Retrospective historical NR cohort	Narrative report	Synoptic note developed by the local cancer surgery working group	40	40	Completeness of operative reports across set of variables developed by the local cancer surgery working group	
Robertston 2020 [17]	Rectal cancer resections	Prospective SR data collection Retrospective historical NR cohort	Narrative report	WebSMR^c^ generated synoptic note	40	40	Completeness of documentation of quality indicators based on articles by McGory et al. [25] and Gagliardi et al. [26]	Time to abstract data from the reports Inter-rater reliability

^a^*BCCA* Binational Colorectal Cancer Audit

^b^*ASCRS* American Society of Colon and Rectal Surgeons

^c^*WebSMR* Web Surgical Medical Record

### Intervention

None of the studies provided a specific definition of NR. All studies, except Kanters et al. [4], used retrospectively collected NR as the comparison group before implementing SR.

Different SR approaches were employed in the included studies. Three studies examining rectal cancer resections developed their SR based on the American Society of Colon and Rectal Surgeons (ASCRS) rectal cancer checklist [3, 7, 16]. Two studies used the Web Surgical Medical Record (WebSMR) program report [17, 18]. Kanters et al. [4] defined SR as an operative note that included any synoptic template within the report. Finally, three studies utilised expert consensus or a surgical working group to develop their SR [2, 12, 16]. Most studies implemented SR using an electronic medical record (EMR), while Bidwell et al. [7, 16] allowed surgeons to dictate synoptic components if an EMR option was not available. Surgeon education before SR implementation was not provided in any study.

### Completeness

Seven studies reported on the completeness of SR compared with NR, assessing various predefined variables. These variables differed among studies, as outlined in Table 1. The mean percentage of included elements detected in SR and NR is summarized in Table 2. All studies showed significantly higher reporting of identified variables in SR compared to NR (*P* < 0.001). Although a meta-analysis was intended, only one study reported the standard deviation of their data, and attempts to obtain missing statistics from authors to impute this data were unsuccessful. Considering the consistent and significant differences across the studies, meta-analysis was deemed unlikely to change the direction of effect.

**Table 2:** Summary of the data regarding completeness of reporting in NR compared with SR

Article	NR	SR	NR MEAN % included elements	SR MEAN % included elements	*P* value
Kanters 2020 [4]	48	62	39	92	*P* < 0.001
Bidwell 2020 [7]	180	118	27	95	*P* < 0.001
Buchanan 2022 [2]	104	491	-	-	*P* < 0.001
	74 colon	347 colon	43	84	*P* < 0.001
	30 rectal	144 rectal	40	84	*P* < 0.001
Maniar 2014 [18]	80	80	24	49	*P* < 0.001
Edhemovic 2004 [12]	40	40	46	99	*P* < 0.001
Maniar 2015 [3]	97	97	32	71	*P* < 0.001
Robertson 2020 [17]	40	40	24	54	*P* < 0.001

### Time to completion

Bidwell et al. [16] conducted qualitative surveys to assess surgeons’ opinions on SR implementation. Some surgeons found SR “quick to complete”, whilst others perceived additional documentation as time-consuming, especially when NR were also required. Edhemovic et al. [12] measured the time needed to complete SR. Excluding the learning curve reports, the mean time for completion was 5 min and 59 s. The time for completion of NR was not reported.

### Surgeon satisfaction

Bidwell et al. [7, 16] explored surgeon satisfaction. Bidwell et al. [7] found that surgeons believed that SR would help capture critical information in their reports, and they identified multiple uses for SR, including quality assurance, improvement, research, and multidisciplinary team communication. Bidwell et al. [16] reported high usability, feasibility, and acceptability of implemented SR, including satisfaction across these domains.

### Educational value

Educational value was mentioned in only one study. Bidwell et al. [7] found that surgeons perceived SR as a tool that could focus their attention on critical element of the operation, serving as a learning or teaching tool.

### Research value

Three studies reported data extraction time and inter-rater agreement for NR and SR.

Maniar et al. [18] demonstrated significantly faster data extraction with SR compared with NR (2:32 min vs 4:01 min, *P* < 0.001). The interrater agreement was also significantly higher for SR than NR (*P* < 0.001).

Maniar et al. [3] demonstrated significantly faster data extraction with SR compared with NR (2:45 min vs 4:48 min, *P* < 0.001). They also found significantly higher inter-rater agreement with SR (*P* < 0.001).

Roberston et al. [17] reported significantly faster abstraction from SR compared to NR (3:46 min vs 6:21 min, *P* < 0.005).

Bidwell et al. [16] highlighted that SR implementation facilitated research by enabling easy data collection, accurate data, and faster analysis. Bidwell et al. [7] found that surgeons believed that the data in SR could provide opportunities for quality assurance, quality improvement, and contribute to research.

### Barriers to implementation

Bidwell et al. [16] identified several barriers to SR implementation. Surgeons expressed frustration when SR integration into an EMR was not feasible, requiring alternative approaches. Technical and administrative oversight for implementation was lacking in some centres, and additional administrative burden, time pressures, and workflow impact were perceived as adverse effects by surgeons.

## Discussion

This systematic review is the first to compare the performance of the SR against the NR in CRC surgery across the domains of completeness, time to complete, surgeon satisfaction, research, and educational value.

The primary outcome of this review focused on the completeness of reporting in SR compared to NR. The included studies consistently demonstrated that SR resulted in improved completeness of reporting of the critical elements of colorectal cancer surgery. This finding is consistent with previous studies assessing SR in different surgery types [8, 9]. Standardised reporting enhances the clarity and readability of reports, reducing ambiguity, and improving communication and collaboration with the multidisciplinary care team. It ensures consistent documentation of key procedural details, such as tumour characteristics, surgical techniques, and perioperative management. The increased completeness provided by SR has significant implications for data extraction, communication within multidisciplinary teams, postoperative care, long-term follow-up, and identification of best practices.

Although only one study assessed the time to completion of SR, conflicting opinions among surgeons were reported. Some surgeons perceived SR as time-efficient, while others felt that the addition of SR to a NR increased the time required to complete the report. However, with careful development, the SR could potentially serve as an alternative to the bulk of the NR, simplifying report interpretation and reducing completion time. Familiarity with the SR components and optimisation of reports with auto-population features may further expedite data entry [2]. Bertlet et al. demonstrated the potential to utilise machine learning and convolutional neural networks to populate SR from the operative video [19]. Additionally, cost savings related to reduced typing time (if reports were dictated) may be achieved with SR [10, 12].

Qualitative studies included in this review revealed that surgeons expressed satisfaction with the use of SR and recognised their value. This positive attitude may be influenced by the American College of Surgeons (ACS) endorsement of SR in CRC surgery as part of its Cancer Surgery Standards Program [20]. The ACS views SR as integral to improving standards in CRC surgery and enhancing adherence to evidence-based guidelines, ultimately leading to improved outcomes [21, 22].

Although limited in this review, studies in other surgery types have highlighted the potential educational benefits of SR [2, 11]. SR can improve trainees’ understanding of critical components of operations and support their comprehension of preoperative decision-making [2, 11].

The structured format of SR facilitates reliable and efficient data collection of key surgical steps and quality indicators for research and clinical registries. Integration into EMR systems enables automated data extraction, reducing costs, and personnel requirements for research [18]. Use of SR reduces potential issues from data managers retrospectively extracting data, improving prospective documentation of quality data as a component of surgical workflow. Future artificial intelligence advancements, such as natural language processing, machine learning, and computer vision, hold promise for automating data extraction and analysis from operative reports, supporting evidence-based decision-making, outcome prediction, and benchmarking across institutions [23, 24].

Although not explicitly mentioned in the included studies, SR may also contribute to robust quality assurance and auditing processes in colorectal surgery. By capturing standardized data elements, operative reports can be easily analysed to assess adherence to evidence-based guidelines, identify variations in practice, and implement targeted interventions for quality improvement [3, 7].

Implementing SR in CRC surgery may encounter challenges related to resistance to change, time constraints, and compatibility with existing electronic health record (EHR) systems. Addressing these challenges requires educational interventions to emphasise the value of SR and provide training on their effective use [1, 7]. Development efforts should focus on user-friendly reports that do not add additional time burden to busy schedules [1, 7]. The nuances of operative findings traditionally communicated in NR should also not be lost. Furthermore, seamless integration of these systems into pre-existing EMRs is critical but may involve substantial work and financial costs for healthcare networks.

### Limitations

This study has limitations, including the small number of studies exploring SR in CRC surgery and limited representation of colon cancer surgery. Heterogeneity exists regarding the elements and quality indicators used to assess completeness of operative report and the specific SR templates employed. Most studies relied on retrospective comparisons with NR, potentially influencing the completeness of NR.

The future of SR in CRC surgery lies in artificial intelligence advancements such as natural language processing, machine learning and computer vision. These technologies hold promise in automating data extraction from operative reports, streamlining documentation processes, and enhancing data utilization for clinical decision support and research purposes. More immediate benefits of standardisation of data collection include enabling bulk upload into national registries such as the National Bowel Cancer Audit (NBOCA) in the United Kingdom and the Bowel Cancer Outcomes Registry (BCOR) in Australia and New Zealand, enhancing audit and research potential.

## Conclusions

This systematic review provides valuable insights into the benefits and challenges associated with the implementation of SR in CRC surgery, highlighting the potential for improved patient and research outcomes. SR offer a standardized and efficient approach to surgical documentation. They contribute to improved communication, enhanced patient outcomes, quality assurance, and research endeavours. Addressing the challenges associated with implementation and harnessing the potential of emerging technologies will further optimize the utility of synoptic operative reports, leading to continued advancements in surgical practice and patient care.
